# A Free, Open-Source, Offline Digital Health System for Refugee Care

**DOI:** 10.2196/33848

**Published:** 2022-02-11

**Authors:** Henry Ashworth, Senan Ebrahim, Hassaan Ebrahim, Zahra Bhaiwala, Michael Chilazi

**Affiliations:** 1 Department of Medicine Harvard Medical School Boston, MA United States; 2 Hikma Health San Jose, CA United States; 3 Department of Public Policy Harvard Kennedy School Boston, MA United States; 4 Johns Hopkins Hospital Baltimore, MD United States

**Keywords:** electronic health record, mHealth, refugee, displaced population, digital health, COVID-19, health care

## Abstract

**Background:**

Rise of conflict, extreme weather events, and pandemics have led to larger displaced populations worldwide. Displaced populations have unique acute and chronic health needs that must be met by low-resource health systems. Electronic health records (EHRs) have been shown to improve health outcomes in displaced populations, but need to be adapted to meet the constraints of these health systems.

**Objective:**

The aim of this viewpoint is to describe the development and deployment of an EHR designed to care for displaced populations in low-resource settings.

**Methods:**

Using a human-centered design approach, we conducted in-depth interviews and focus groups with patients, health care providers, and administrators in Lebanon and Jordan to identify the essential EHR features. These features, including modular workflows, multilingual interfaces, and offline-first capabilities, led to the development of the Hikma Health EHR, which has been deployed in Lebanon and Nicaragua.

**Results:**

We report the successes and challenges from 12 months of Hikma Health EHR deployment in a mobile clinic providing care to Syrian refugees in Bekaa Valley, Lebanon. Successes include the EHR’s ability to (1) increase clinical efficacy by providing detailed patient records, (2) be adaptable to the threats of COVID-19, and (3) improve organizational planning. Lessons learned include technical fixes to methods of identifying patients through name or their medical record ID.

**Conclusions:**

As the number of displaced people continues to rise globally, it is imperative that solutions are created to help maximize the health care they receive. Free, open-sourced, and adaptable EHRs can enable organizations to better provide for displaced populations.

## Introduction

There are over 80 million displaced people worldwide, and this number is projected to rise with increasing rates of natural disasters, conflict, and infectious disease outbreaks [1]. Over the last year, natural disasters and conflicts have been compounded by the impact of the COVID-19 pandemic. Displaced populations have been significantly affected due to limited ability to implement social distancing measures and minimal access to vaccines [2]. At baseline, displaced populations face unique health threats, ranging from violence to food insecurity, infectious diseases, and exacerbation of underlying chronic conditions [3].

Limited resources and continual displacement create unique challenges for effective health care delivery to refugees, particularly when superimposed on the burdens of chronic diseases such as diabetes and hypertension [2]. When it comes to meeting these challenges, health care organizations caring for displaced populations often operate without formal systems for maintaining records of patient information. The lack of formal record-keeping can create even more challenges in establishing regular and consistent care [3]. This lack of consistency particularly impacts the continuity of care for displaced persons with chronic diseases. Overall, disjointed systems lead to an increased patient burden and perpetuate poor health outcomes [4].

A systematic review showed that general health records improved health outcomes in refugee populations [5]. Although electronic health record (EHR) systems have traditionally been built for high-resource settings optimized for billing, they have the unique ability of being adaptable to meet the needs of health care settings serving displaced populations. Previous research on implementing EHRs in displaced populations has shown that they lead to better patient outcomes through tracking of disease markers, increasing provider adherence to guidelines, and increasing patient adherence [4,6-10]. However, there are important barriers to effectively implementing EHRs in displaced population settings. In particular, EHRs need to be adaptable, and providers need to be engaged to drive overall uptake and success [6,7,11].

With this foundation in mind, we started a nonprofit organization, Hikma Health, to develop an EHR system to meet these needs and advance the care provided for displaced populations. Hikma Health was first started as an initiative from the Massachusetts Institute of Technology (MIT) Media Lab’s 2017 Refugee Learning Accelerator. During a visit with refugee youth and their caregivers in Amman, Jordan, we realized the need for a free and open-source EHR system to provide continuity in their care. We incorporated as a 501(c)(3) nonprofit organization in California. Our initial seed funding came from the Harvard Business School New Venture Competition, and we have since grown with the support of private philanthropic foundations.

## Methods

### EHR Design Methodology

We refocused EHR development by employing a human-centered design approach that has been used successfully in global health technology, particularly for chronic disease management [12]. We started by conducting 30 patient and 12 provider interviews in Jordan, Lebanon, Turkey, and Greece. These interviews included a variety of displaced population health care settings, including mobile clinics, multispecialty clinics, and hospitals, to identify current gaps in documentation and needs to improve care through desired workflows. Additionally, we collected documentation templates reflecting providers’ then-current paper-based health record-keeping in Lebanon. We observed the clinician practice at five clinical practices in both rural and urban settings in Lebanon and Jordan. We also conducted three focus groups on the preliminary design concept with providers of different specialties, including internal medicine, nursing, neurology, cardiology, pediatrics, dentistry, mental health, and social work. The results from the interviews and focus groups were analyzed using a framework analysis. We designed the architecture of the Hikma Health system and prototyped it with the early support of these providers and patients.

### Hikma Health Design Features

**Figure 1:**
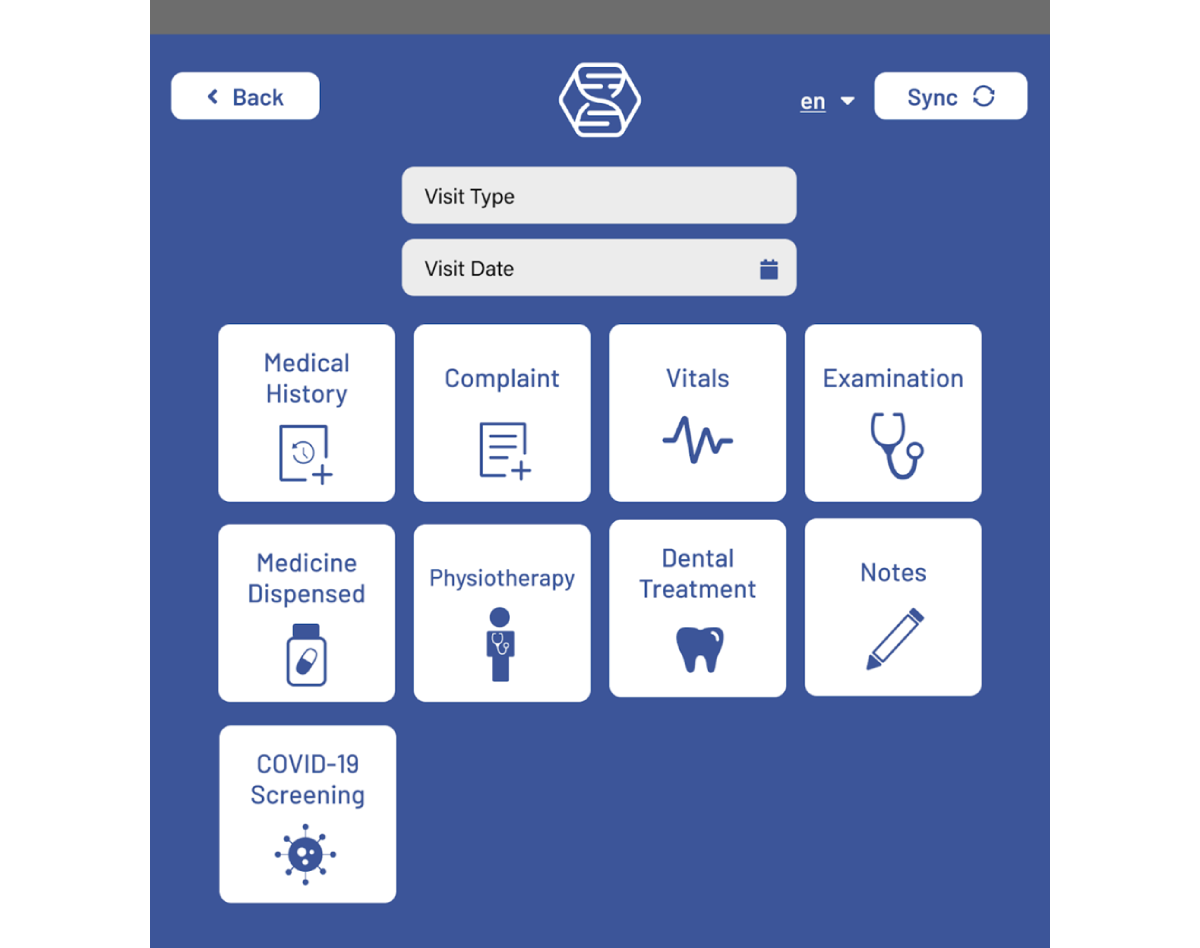
New Visit dashboard showing different modular workflows that can be modified for a clinic’s needs.

**Figure 2:**
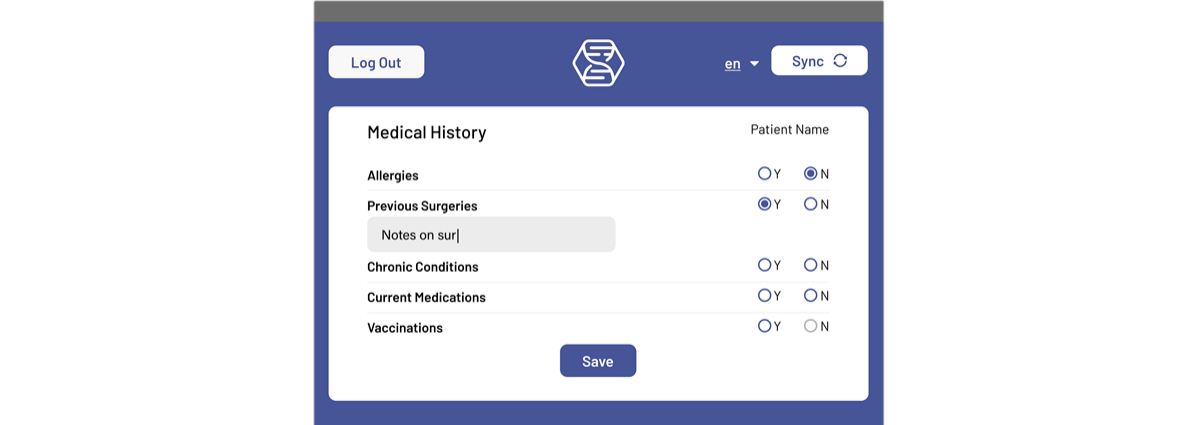
An example of the “medical history” modular workflow that can be simply filled out and modified.

Through our human-centered design process, we identified three essential features for an effective digital health system in low-resource settings: modular workflows, multilingual interfaces, and offline-first capabilities. We then designed the Hikma Health app as an offline-first multilingual mobile EHR system with 32 modular workflows and sensible defaults for the global care of mobile populations, including a clinically validated COVID-19 screening tool. Although first-generation EHRs were limited by technical constraints [13], modern EHRs such as the Hikma Health system incorporate modular workflows. This modularity is essential to appropriately provisioning the app based on local clinical practice, such as consulting specialty care services or tracking prescriptions over time [14]. Modular workflows refer to distinct premade documentation forms that can be included in a particular EHR deployment based on services available at that clinic. Working within this modular framework enables clinics to deploy a functional system rapidly by provisioning the relevant workflows as plug-and-play modules (Figure 1). Each module is further modifiable, offering full customizability as needed to optimize care (Figure 2).

In light of the well-documented challenges for multilingual care practices in refugee care settings, we built the Hikma Health app to fully support English, Arabic, and Spanish with support for instantaneous translation for multilingual provider teams. In addition, recent advances in database technology and smartphone hardware enabled us to provision the Hikma Health system as a functional offline-first app with automatic data synchronization to a central server whenever a mobile device is connected. An offline-first capability continues to be essential in many remote areas where displaced populations receive care; however, most EHR systems designed for the developed world do not have this capability.

### Sustainability

Hikma Health initially supported the deployment of the Hikma Health system directly by providing cloud storage and mobile devices, employing US-based technical staff to customize the deployment, and supporting local engineers in-country to take over the deployment. This model was optimal for the early development and optimization of the software system in 2018-2020. In 2021, we launched the Year of Migrant Health program to support independent deployment of the stable release of the Hikma Health system at a wider, global scale. In this grant program, we are granting up to US $25,000 per clinical organization to offset costs of independent deployment, including cloud storage, mobile devices, and engineering staff costs. We believe the Year of Migrant Health deployment model will expand the use of the Hikma Health system, strengthen its open-source modular workflow library as developers contribute globally, and demonstrate a sustainable means for clinics to autonomously deploy their EHRs.

### Deployment

The open-source Hikma Health system is freely available to organizations with the technical capacity to self-deploy the platform on a cloud server. However, many clinical organizations working in low-resource settings lack the technical expertise and resources to self-deploy. Therefore, our technical team works directly with clinical partners to both manage deployment and customize our generic EHR system to fit their specific medical workflows.

The Hikma Health system is currently in use by two clinical partners, Endless Medical Advantage (EMA) and Nueva Vida Clinic. EMA is a mobile health clinic based in Bekaa Valley in Lebanon that travels throughout the region serving hundreds of informal and dispersed Syrian refugee camps, as well as vulnerable local communities. EMA has been actively using the Hikma Health system since September 2020. Nueva Vida Clinic in Ciudad Sandino, Nicaragua, is a primary health care facility that provides free and subsidized care to rural and low-income communities, including migratory populations. Nueva Vida Clinic just started actively using the Hikma Health system since September 2021. We have been assessing the strengths and limitations of the Hikma Health system through monthly quality assurance and quality improvement meetings with our partners.

### Ethical Considerations

A formal ethical approval for this study was not sought as it was a general inquiry concerning EHR perspectives and by the Harvard Longwood Campus Institutional Review Board it did not meet the US federal definition for research.

## Results

### Reported Outcomes

In the 12 months that EMA has been using the Hikma Health system, they have been able to transition from a paper record–based system of patient health data collection to a digital system. EMA health care workers have reported that using the Hikma Health system has increased the efficiency with which they are able to collect and access patient health information, particularly in the field while seeing patients. Previously, EMA reported that they had been unable to see past the visit history of a particular patient, but with the Hikma Health system were able to view all previously collected health information, including past diagnoses and prescriptions that were of particular importance. Additionally, EMA physicians and the administration have specified that the offline functionality of the system was essential for their operations, given the limited network connectivity throughout Bekaa Valley.

The customizability of the Hikma Health system also enabled continuous improvement to the medical forms and workflows throughout the past year, enabling the Hikma Health team of developers to easily adapt the system to fit EMA’s dynamic needs. For example, EMA was able to deploy a screening module for COVID-19 to easily identify high-risk patients for isolation and testing.

Prior to using the Hikma Health system, EMA administrative staff would have to manually process paper records to compile key organizational metrics of the organization, such as total patient visits, prevalence of particular diagnoses, and number of medications prescribed. These organizational metrics are essential to the clinic’s programmatic efficiency and operations. With the Hikma Health system’s administrative dashboard, which allows administrative staff to easily export all of the clinic’s patient data, administrative staff reported that they were able to track outcomes of the clinic with greater ease compared to the paper-based system.

As Nueva Vida Clinic has just started to use the Hikma Health system, we do not have any reported results from the field. However, important lessons were learned from the customization of the Hikma Health system to the needs of Nueva Vida Clinic.

### Technical Issues and Lessons Learned

One of the challenges that arose during the deployment with EMA surrounded the ability to easily search and filter for individual patients. Because many Arabic names are transliterated in multiple ways with different spellings (ie, Muhammad or Muhamad), clinician users were having a hard time finding the correct identity in an efficient manner. Although they were able to find patients using additional search parameters such as date of birth, phone number, or hometown, clinician users expressed frustration at the challenges with using a first-name search for many patients. In response, we implemented a new feature within the Hikma Health system that allowed for “fuzzy searches,” which would include all variations of the spelling of the most commonly occurring names within the population. Although this feature required manually compiling this list of names and possible spellings, upon implementation, it dramatically improved the efficiency of the search feature within the field.

Another technical challenge we encountered during the deployment with EMA was an issue with the syncing feature of the system when certain fields within the patient registration were missing. The backend database of the system generated a unique user identification number as a combination of digits including a patient’s date of birth. If during patient registration, the date of birth was not collected by the clinician user, the unique user identification number would be unable to be generated, creating an error when trying to sync the data. This issue prevented successful synchronization for multiple days before being noticed by the clinical team. In response, we pushed out a software fix that resolved the issue of the unique user identification number not being created for patients with a missing date of birth. Furthermore, we created an alert with the mobile app that notified clinician users of the status of the sync, either confirming its success or flagging its failure.

## Discussion

### Principal Findings

As natural disasters, conflicts, and infectious disease outbreaks continue to increase the number of people displaced worldwide, innovations are needed to meet their needs and the limitations of settings in which health care is provided. EHRs are one possible solution, yet they must be adaptable and implemented with strong provider uptake and buy-in [6,7,11]. By taking a user-centered design approach, we have created an EHR that meets many of these needs, including offline capabilities for areas without internet connection, modular workflows to simplify the user interface, and multilingual capability to increase accessibility. Compared to other EHRs, our system is fully open-sourced [7,10,15]. This allows any use with the support of a software engineer to adapt the Hikma Health EHR platform to meet a clinic’s needs. Our EHR also has a few limitations, including no direct way for patients to carry a copy of their own medical record, an innovative feature introduced by the Sijilli EHR created by Epic [15]. Although the Hikma Health EHR platform does not contain every element to meet a health system’s needs out of the box, this adaptability is a central feature allowing for customization.

From our informal results to date, we have seen the Hikma Health system meet the needs of two different health care organizations. In the remote areas of Bekaa Valley to rural Nicaragua, our partners have been able to adapt the Hikma Health system to meet the constraints of their environment while still providing for their patients. To continue to ensure sustainability of the Hikma Health system, we support the hiring and training of in-country software engineers to address bugs or updates our partners require.

The biggest limitation of this system is that it may not be perfectly suited for every clinic once downloaded. As each health care setting caring for displaced populations is different, we cannot create an EHR to meet every need. Additionally, it does take time and resources for a health care setting to actually set up an EHR. These resources include hardware and technical personnel. As previously mentioned, an EHR is not a solution within itself. Finally, to be successful, any EHR requires engagement of all its users from health care providers to administrators. Therefore, each EHR, including our own, requires early and continual engagement to ensure long-term success.

Looking to the future, it is clear from research to date that EHR innovations for displaced populations offer a means to improve care [4-10]. Future innovations should continue to take user-centered design approaches and consider such factors as provider-tailored modular workflows and offline capabilities. Additionally, future innovations should consider ways to expand patient access to their medical records, ease data transfer across systems or organizations, integrate diagnostic technologies, and decrease technical barriers to implementation.

### Conclusions

As displaced people around the world continue to face the COVID-19 pandemic and other health care challenges, it is imperative that adaptable EHR solutions are developed to meet their specific health needs. The Hikma Health system is a free EHR optimized for mobile, offline use that has been designed with the user in mind to meet the needs of displaced patients. By making the system free and open source, we aim to enhance every organization’s capacity to provide better care for displaced populations worldwide.

## Abbreviations

EHR: electronic health record
EMA: Endless Medical Advantage
MIT: Massachusetts Institute of Technology
